# Increased KCNQ3 expression in papillary thyroid cancer promotes proliferation and migration

**DOI:** 10.1186/s12935-025-04049-6

**Published:** 2025-11-17

**Authors:** Qiuli Li, Muyuan Liu, Xuhong Song, Lingzhu Xie, Dongchen Liu, Ting Su, Yangzhan Xu, Gengquan Li, Bin Liang, Dongyang Huang

**Affiliations:** 1 Department of Central Laboratory, Cancer Hospital of Shantou University Medical College, Shantou, China; 2 Department of Cell Biology and Genetics, Key Laboratory of Molecular Biology in High- Cancer Incidence Coastal Chaoshan Area of Guangdong Higher Education Institutes, Shantou University Medical College, No. 22, Xinling Road, Shantou, 515041 China; 3 Department of Head and Neck, Cancer Hospital of Shantou University Medical College, Shantou, China; 4 Department of Radiotherapy, Cancer Hospital of Shantou University Medical College, No. 7 Raoping Road, Shantou, 515041 Guangdong China

**Keywords:** ESR1, KCNQ3, Papillary thyroid cancer, RAS/RAF/MAPK signaling pathway, XE991

## Abstract

**Purpose:**

Papillary thyroid cancer (PTC), the most prevalent thyroid malignancy, is witnessing a global surge in incidence. The potassium voltage-gated channel subfamily Q member 3 (KCNQ3) is aberrantly overexpressed in PTC, yet its mechanistic contribution to oncogenesis remains unclear. Thus, we aimed to elucidate the oncogenic mechanism of KCNQ3 in PTC.

**Methods:**

We integrated gene expression profiling interactive analysis (GEPIA), immunohistochemistry, and western blotting to assess KCNQ3 expression during PTC tumorigenesis and progression, and validated in vitro and in vivo using BALB/c nude mice. The functional roles of KCNQ3 were evaluated using wound-healing, transwell, and colony formation assays. Protein interactions were elucidated through co-immunoprecipitation, mass spectrometry (MS), and immunofluorescence, while estradiol (E_2_)-mediated KCNQ3 regulation was examined using chromatin immunoprecipitation–qPCR (ChIP–qPCR). The therapeutic potential of the KCNQ channel inhibitor, XE991, was also investigated.

**Results:**

KCNQ3 was upregulated in PTC and drove tumor cell proliferation and migration. Mechanistically, KCNQ3 interacted with GRB2-associated regulator of MAPK1 subtype 1(GAREM1), growth factor receptor-bound protein 2(GRB2), and SOS Ras/Rac guanine nucleotide exchange factor 1 (SOS1), activating the RAS/RAF/MAPK signaling cascade to promote oncogenesis. ChIP–qPCR revealed that E_2_ enhanced KCNQ3 transcription by binding estrogen receptor alpha (ESR1) to the KCNQ3 promoter. Notably, XE991 inhibited PTC cell proliferation and migration.

**Conclusion:**

Our research uncovers a novel KCNQ3-driven oncogenic axis in PTC, establishing KCNQ3 as a promising therapeutic target. Our findings also establish E_2_ as a KCNQ3 regulator in PTC, elucidating a mechanism underlying the female gender bias of the disease. Additionally, XE991 shows potential in PTC treatment.

**Supplementary Information:**

The online version contains supplementary material available at 10.1186/s12935-025-04049-6.

## Introduction

Papillary thyroid cancer (PTC), comprising 80–85% of all thyroid malignancies [1, 2], is the predominant form of thyroid cancer, with a global incidence that has surged by 62% from 2003 to 2017, rising from 9.9 to 16.1 cases per 100,000 individuals [3–5]. Despite its generally favorable prognosis, PTC carries a 20–30% recurrence rate, significantly elevating mortality risk [1, 2, 6]. Traditionally, the pathogenesis of PTC, involves a highly complex and interconnected network of cellular and molecular events that drive its initiation and, crucially, the development of aggressive phenotypes. While activating mutations in classical oncogenes such as BRAF and RAS are fundamental initiating drivers [3], their oncogenic effects are exerted primarily through aberrant and constitutive activation of key signaling pathways, most notably the MAPK (mitogen-activated protein kinase), PI3K/AKT cascades [3, 7], and JAK/STAT3 pathway [8], which are critical for sustained proliferation and anti-apoptotic signaling. The oncogenic and metastatic potential of PTC is driven by cellular plasticity through epithelial-mesenchymal transition (EMT), tumor cell metabolic programming, epigenetic alterations, and tumor microenvironment (TME) remodeling [3]. These non-mutational drivers, involving metabolic shifts, signaling pathway dysregulation, and paracrine signaling, promote malignancy, immune evasion, drug resistance, and metastatic niche formation, necessitating a comprehensive understanding for targeted therapies [3].

However, our research has suggested that a new trigger, the potassium voltage-gated channel subfamily Q member 3 (KCNQ3), activates a novel signaling axis. KCNQ3, a 672-amino acid protein, forms heteromeric ion-conducting channels with potassium voltage-gated channel subfamily Q member 2 (KCNQ2), generating an M-current to stabilize neuronal excitability [9–15]. While historically studied in cardiac and neural contexts [16–18], emerging evidence implicates KCNQ family members in oncogenesis, notably in gastrointestinal malignancies, where KCNQ3 promotes epithelial-mesenchymal transition and tumor proliferation via Wnt signaling [19, 20]. The pharmacological inhibition of KCNQ3 has shown promise in attenuating cancer cell growth in these contexts [19]. In PTC, KCNQ3 has emerged as a hub gene associated with lymph node metastasis and recurrence, with transcriptomic analyses revealing its robust upregulation in tumor tissues and cell lines, including B-CPAP and TPC-1, compared to normal thyroid epithelium [21, 22]. However, the functional roles and regulatory mechanisms driving KCNQ3 overexpression in PTC remain unknown.

The striking female predominance in PTC, illustrated by the 1:3 male-to-female incidence ratio [25–28], prompted us to hypothesize that estrogen signaling, mediated by E_2_, may underpin KCNQ3 upregulation in some female patients. Additionally, the KCNQ channel blocker XE991, known for targeting KCNQ2/KCNQ3 channels in neurological disorders [29–31], offers a potential therapeutic avenue. Here, we investigate the mechanistic contributions of KCNQ3 to PTC progression, elucidate the possible role of E_2_ in its dysregulation, and evaluate XE991 as a candidate therapy, aiming to bridge critical gaps in PTC biology and treatment.

## Materials and methods

### Cell lines

Human PTC cell lines (KTC-1, B-CPAP, and TPC-1) and a human thyroid follicular epithelial cell line (Nthy-ori3-1) were purchased from the National Cell Culture Centre (Shanghai, China). This study only used cell lines that had been passed for a maximum of 20 generations. All cell lines were cultured in RPMI 1640 medium (RPMI 1640; Gibco, USA) supplemented with 10% fetal bovine serum (FBS; Gibco, USA), in an incubator at 37 °C, and 5% CO_2_. The three human PTC cell lines utilized in this study—B-CPAP, TPC-1, and KTC-1—were specifically chosen to represent the major molecular subsets of PTC, thus ensuring the generalizability of our mechanistic findings. B-CPAP harbors the most prevalent oncogenic mutation, BRAFV600E, TPC-1 is driven by RET/PTC1 rearrangement, and KTC-1 typically exhibits a RAS-like profile (often carrying RAS or TERT promoter mutations), representing the non-BRAF/RET subtype. The B-CPAP cell line was derived from the tumor of a woman who developed poorly differentiated thyroid cancer. KTC-1 cells were isolated from a pleural effusion metastatic lesion in a male patient with poorly differentiated thyroid cancer. The TPC-1 cell line was derived from a differentiated thyroid cancer in a Japanese adult woman with PTC.

### Cell transfection

To knock down human KCNQ3 and ESR1, shRNA sequences targeting KCNQ3 or ESR1 were inserted into an LV3 (H1/GFP ± puro) lentiviral cloning vector (Guangzhou IGE Biotechnology Co., Ltd., Guangzhou, China). Table S3 lists the shRNA sequences used in this study. The virus was prepared in HEK293T cells transfected with lentiviral packaging vectors via FuGENE (Promega Biotech Co., Ltd., Beijing, China), and used to infect TPC-1 and B-CPAP cells. We purchased premade lentivirus encoding of KCNQ3 with a co-expressed green fluorescence protein (Guangzhou IGE Biotechnology Co., Ltd. Guangzhou, China), and transduced KTC-1 and B-CPAP cells. Transduced cells for knockdown or overexpression were selected using puromycin (2 µg/mL) for 2 weeks, after which protein knockdown or overexpression was confirmed via western blotting.

### Immunohistochemistry

An anti-KCNQ3 primary antibody (1:100 dilution, rabbit, APC-051, Alomone Labs, Ltd. Jerusalem Israel), and mouse anti-estrogen receptor alpha monoclonal antibody (1:100 dilution, MA1-310, Thermo Fisher Scientific) were separately incubated with paraffin-embedded tissue slices overnight at 4 °C, followed by a 30-min incubation with horseradish peroxidase (HRP)-conjugated secondary antibody at room temperature. Slides were then incubated with diaminobenzidine and counterstained with hematoxylin.

### Western blotting

Cells were subjected to lysis in RIPA buffer containing phosphatase inhibitors (Beyotime Biotech). After centrifugation, the supernatant was subjected to BCA protein quantification. The samples underwent denaturation, followed by separation of proteins using 10% SDS-PAGE, and transfer of proteins to polyvinylidene difluoride (PVDF) membranes (Sigma-Aldrich). Membranes were incubated overnight with primary antibody, after which they were thoroughly washed and incubated with secondary antibody for 1 h at 24 °C. Signals were detected using an enhanced chemiluminescence kit, and imaging was performed using a ChemiDoc system. Table S2 lists of the antibodies utilized.

### Wound-healing assay

The wound-healing assay was performed by seeding a 6-well culture plate with 1 × 10^6^ cells and incubating at 37 °C for 24 h. A scratch wound was created using a 20-µL pipette tip, and the growth medium was replaced with RPMI 1640 medium containing 0.5% FBS. High-resolution images of the wound edges were captured at 0, 12, 24, 36, 48, and 60 h at the same location. Image-Pro Plus 6.0 was used to calculate the average distance between the cell edges at each time point to assess the degree of cell migration.

### Colony formation assay

In this assay, each well of a 6-well plate was seeded with 200 cells, followed by incubation for 7 d at 37 °C. The plates were then fixed with 4% paraformaldehyde for 30 min, followed by staining with 0.1% crystal violet (C0121; Beyotime) for 30 min. Before imaging, the plates were rinsed with ddH_2_O and dried.

### Transwell assay

In total, 5 × 10^4^ cells in 200 µL of serum-free RPMI 1640 were seeded in the upper chamber of an 8-µm pore size transwell plate (3422, Corning, USA). Furthermore, 600 µL of RPMI 1640 containing 20% FBS was added to the lower chamber. After allowing cells to migrate through the Matrigel matrix (3445-010-01, R&D Systems) for 24 h, they were fixed with 4% paraformaldehyde for 30 min and stained with 0.1% crystal violet (C0121, Beyotime) for 30 min. Migrated cells were counted in five randomly selected fields of view under a bright-field microscope.

### Xenograft tumor models

BALB/c nude mice (5–6-week-old; male) were procured from Charles River Laboratories (CRL) (Beijing, China) and segregated into distinct groups (7 mice/group): shKCNQ3, scramble, KCNQ3-overexpressing, and vector. Each nude mouse was administered 5 × 10^6^ B-CPAP cells in 100 µL of Matrigel via subcutaneous injection on the back. After 34 d, all mice were euthanized via cervical dislocation, and their tumors excised. All experimental procedures were approved by the Animal Experimental Ethics Committee of Shantou University Medical College (Approval No. SUMC2022-001).

### Co-immunoprecipitation (Co-IP)

Protein-protein interaction was examined via co-IP. Cells were lysed in RIPA buffer supplemented with protease and phosphatase inhibitors (VL312018; Thermo Fisher Scientific, USA). After centrifugation, the protein extracts were pretreated with protein A/G magnetic beads (HY-K0202; MCE, USA) for 2 h. After the magnetic beads were removed, supernatants were incubated with primary antibody or normal rabbit IgG overnight on a rotating platform. The protein extracts were incubated with protein A/G beads on a rotating platform at 4 °C for 6 h, then beads were washed with PBST, and the bound proteins were denatured by boiling in SDS‒PAGE buffer and then separated by 10% SDS‒PAGE for western blotting or LC-MS. Table S2 lists the antibodies used for co-immunoprecipitation.

### Immunofluorescence

Plasmids encoding green fluorescent protein and KCNQ3 were pre-transfected into the KTC-1 and B-CPAP cell lines. PTC cells (1 × 10^5^) were seeded on slides and put into a 6-well culture plate and incubated for 24 h. Subsequently, the cells were fixed in 4% paraformaldehyde for 30 min. The slides were blocked for 30 min, and then incubated overnight at 4 °C with antibodies against rabbit GAREM1 (1:100, PA5-20845, Thermo Fisher Scientific). After rinsing with PBS, anti-rabbit secondary antibodies (A-11012; Invitrogen) at a 1:500 dilution were applied for 1 h at room temperature. Finally, the slides were mounted in an Antifade Mounting Medium with DAPI (P0131–25 ml, Beyotime) and stored at 4 °C. The slides were imaged via a laser scanning confocal microscope (LSM800, Carl Zeiss).

### Chromatin Immunoprecipitation (ChIP)-seq data analysis

ChIP-seq data were used to identify putative promoters and possible binding sites for ESR1 on KCNQ3. ESR1 ChIP-seq data (GSM7299939) [41], and H3K27ac ChIP-seq data for the B-CPAP cell line (GSE120175) [42] were downloaded from the Gene Expression Omnibus database. H3K4me and H3K27ac ChIP-seq data (ID: 1030615) for the TPC-1 cell line were downloaded from the Sequence Read Archive database. ChIP-seq data were processed and visualized using the UCSC Genome Browser.

### ChIP

The ChIP assay was performed using a chromatin immunoprecipitation kit (Sigma‒Aldrich, 17–611) according to the manufacturer’s instructions. Immunoprecipitated DNA was characterized via qPCR and normalized to the input DNA. Antibodies used in the ChIP analysis were mouse IgG and ESR1 antibodies. Table S2 lists the antibodies used for chromatin immunoprecipitation.

The following primers targeting the KCNQ3 promoter were used:

5’-CCTAAGACCTCCGTCCTAGC-3’.

3’-CGCAGCACTCTAATTTGTTACC-5’.

### Quantitative PCR (qPCR)

qPCR was performed using AceQ qPCR SYBR Green Master Mix (Low ROX Premixed) (Vazyme, Q131-02) on a QuantStudio 12 K Flex Real-Time PCR System (Thermo Fisher, Waltham, MA, USA) per the manufacturer’s protocols.

### Gene expression profiling interactive analysis (GEPIA)

Gene correlations between ESR1 and KCNQ3 were analyzed via GEPIA [32]. KCNQ3 gene expression data were analyzed via GEPIA2 (http://gepia2.cancer-pku.cn/) based on The Cancer Gene Atlas (TCGA) and GTEx datasets. Differential gene expression and gene correlation analysis were performed using a cutoff value of |log2FC| >1 and a p-value < 0.05.

### Protein mass spectrometry

KCNQ3 was immunoprecipitated from B-CPAP cells, and immunoprecipitates were digested with trypsin for analysis via LC-MS on a Q Exactive HF system. Peptide identification was performed using Mascot against UniProt with a 1% false discovery rate. Proteins were quantified via label-free quantification.

### Statistical analyses

All statistical analyses were performed via GraphPad Prism (version 8.0) software. The t-test was used to compare two experimental groups. All statistical tests were two-tailed unless otherwise specified.

## Results

### KCNQ3 is upregulated in PTC

**Fig. 1:**
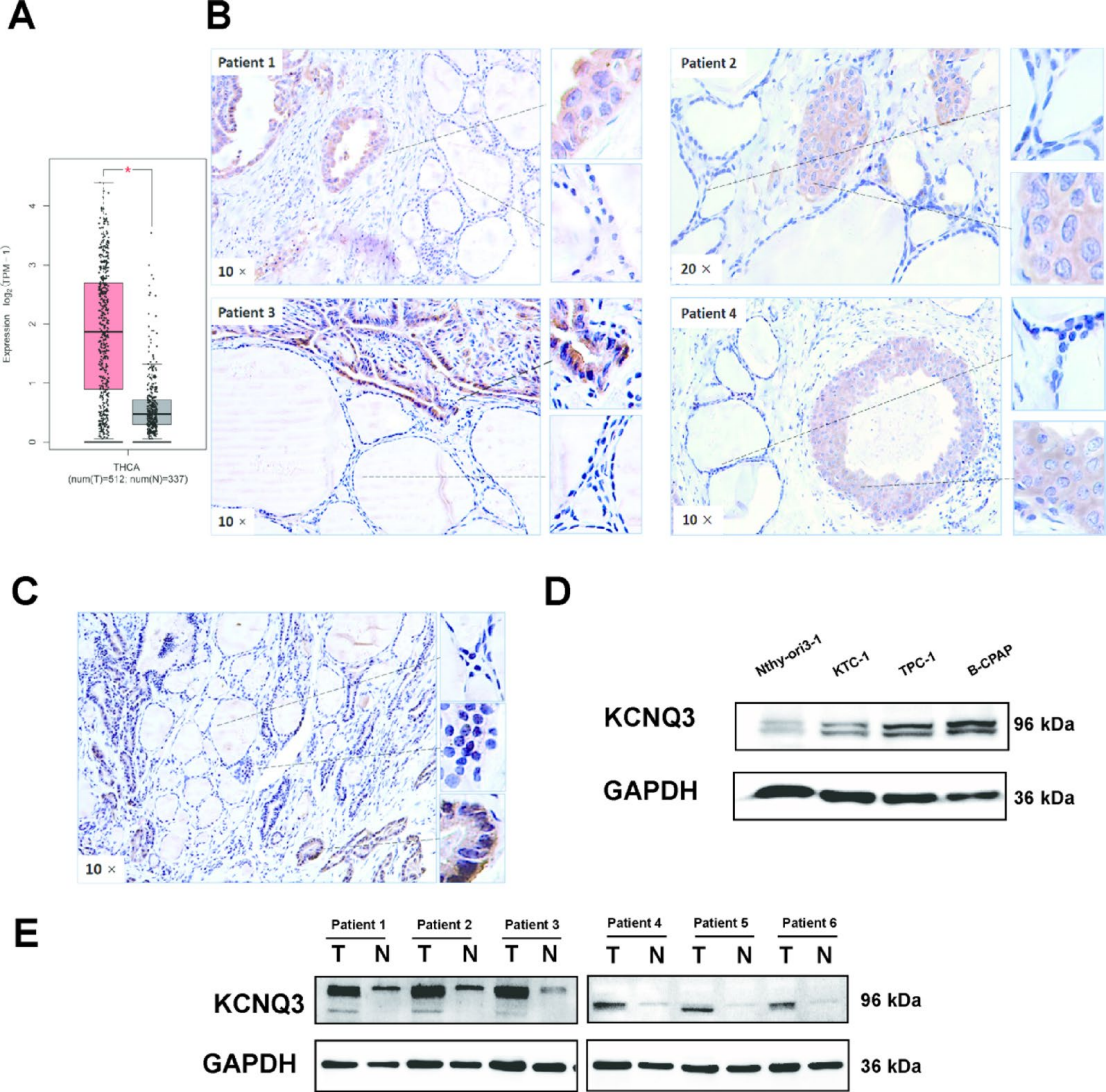
KCNQ3 is upregulated in thyroid cancer (**A**) The Cancer Genome Atlas (TCGA) data analyzed via GEPIA [32] shows KCNQ3 upregulation in thyroid cancer (T: tumor, N: normal). Transcripts per million (TPM) counts per transcript length (kb) per million reads mapped via RNA-seq. (**B**,** C**) Immunohistochemical analysis of PTC tissue shows higher KCNQ3 expression in tumor cells than in normal thyroid follicular epithelial cells adjacent to the tumor. KCNQ3 expression levels are correlated with tumor cell occurrence and deterioration. (**D**) Western blot images show KCNQ3 expression in Nthy-ori3-1 cells and three PTC cell lines. (**E**) Western blot images show higher KCNQ3 expression levels in PTC tissue samples than in normal thyroid tissue samples

To investigate KCNQ3 expression in PTC, we used the GEPIA database, leveraging TCGA data to assess KCNQ3 mRNA levels. This analysis revealed significant KCNQ3 overexpression in PTC compared to normal thyroid tissue (Fig. 1A). To validate these findings and assess clinical relevance, we performed immunohistochemistry (IHC) on 45 paired PTC and adjacent non-tumor thyroid tissue samples. These surgical specimens were obtained from patients at the Cancer Hospital of Shantou University Medical College. IHC demonstrated markedly elevated KCNQ3 protein levels in tumor epithelium relative to adjacent follicular epithelial cells, with 28 of 45 tumors exhibiting moderate to strong staining versus 35 of 45 normal tissues exhibiting weak to moderate expression (Fig. 1B). Notably, within single microscopic fields, KCNQ3 expression displayed a gradient, with intense staining in tumor cells, moderate staining in partially differentiated follicular cells, and minimal staining in normal thyroid epithelium, correlating with tumor progression (Fig. 1C). Further statistical analysis correlating KCNQ3 protein expression levels with clinical features revealed a significant association between KCNQ3 upregulation and PTC development. Specifically, high KCNQ3 expression was found to correlate strongly with gender, positive lymph node metastasis, and larger primary tumor size (Table S1). Western blot analysis further confirmed robust KCNQ3 upregulation in PTC cell lines (KTC-1, TPC-1, and B-CPAP) compared to the non-malignant Nthy-ori3-1 thyroid cell line (Fig. 1D). Consistently, patient-derived PTC tissues exhibited substantial KCNQ3 protein elevation relative to paired healthy tissues (Fig. 1E). These convergent findings establish KCNQ3 overexpression as a hallmark of PTC, closely linked to tumorigenesis and disease progression.

### KCNQ3 promotes PTC proliferation and migration

**Fig. 2:**
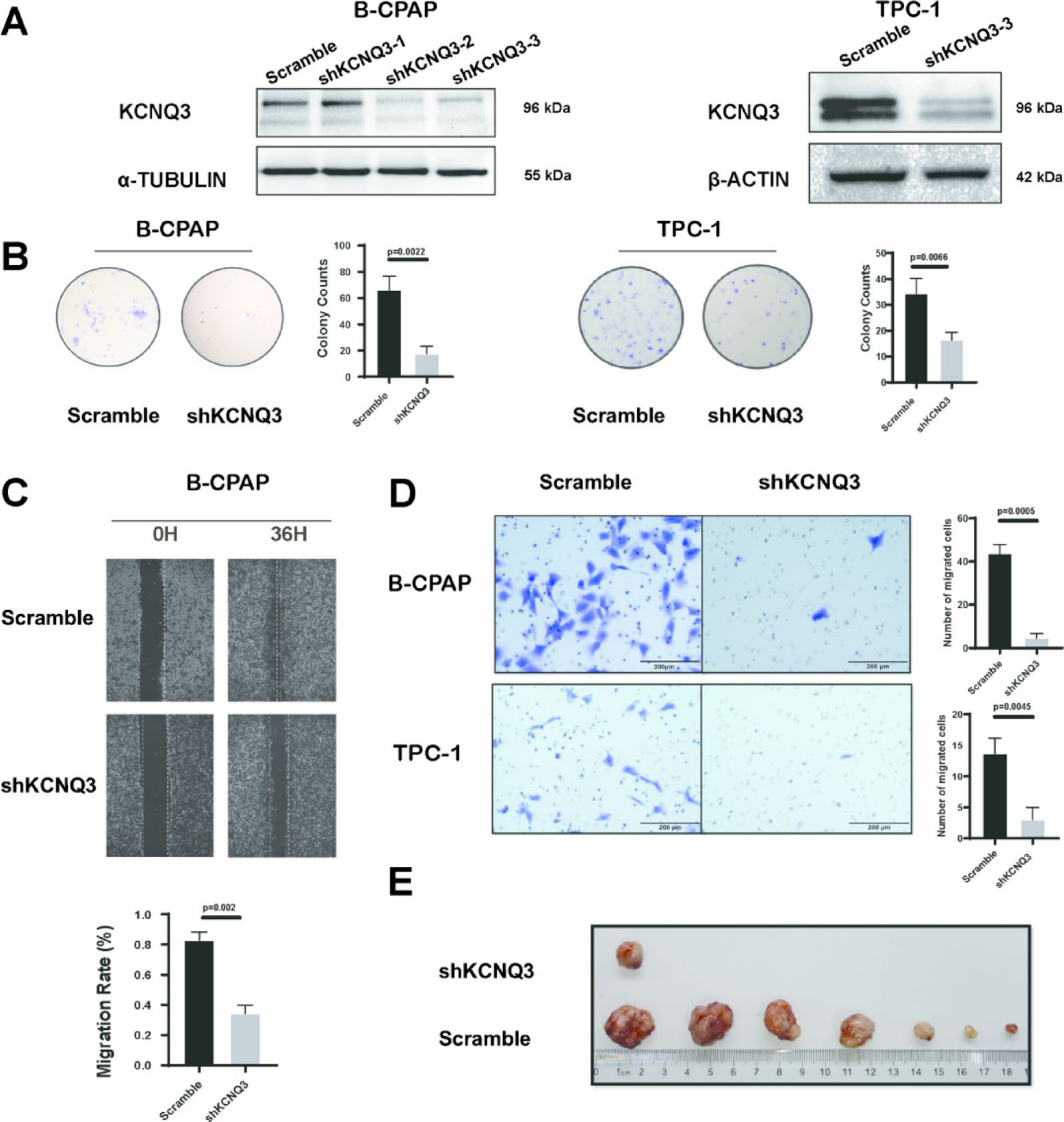
KCNQ3 knockdown inhibits PTC cell proliferation and migration in vitro and *in vivo. *(**A**) Knockdown of KCNQ3 expression in B-CPAP and TPC-1 cells via infection of PTC cells with NC (scrambled shRNA) or shKCNQ3 lentivirus. (**B**) Colony formation assay showing inhibition of cell proliferation after KCNQ3 knockdown in B-CPAP and TPC-1 cells. (**C**) Wound healing assay showing the inhibition of cell migration after KCNQ3 knockdown in B-CPAP cells. (**D**) Transwell assay showing decreased migration and invasion in B-CPAP and TPC-1 cells after KCNQ3 knockdown. (**E**) Images of tumors removed from nude mice. Statistical significance of all the data was determined via two-tailed Student’s t-tests. Data are shown as the means ± S.D., *n *= 3

**Fig. 3:**
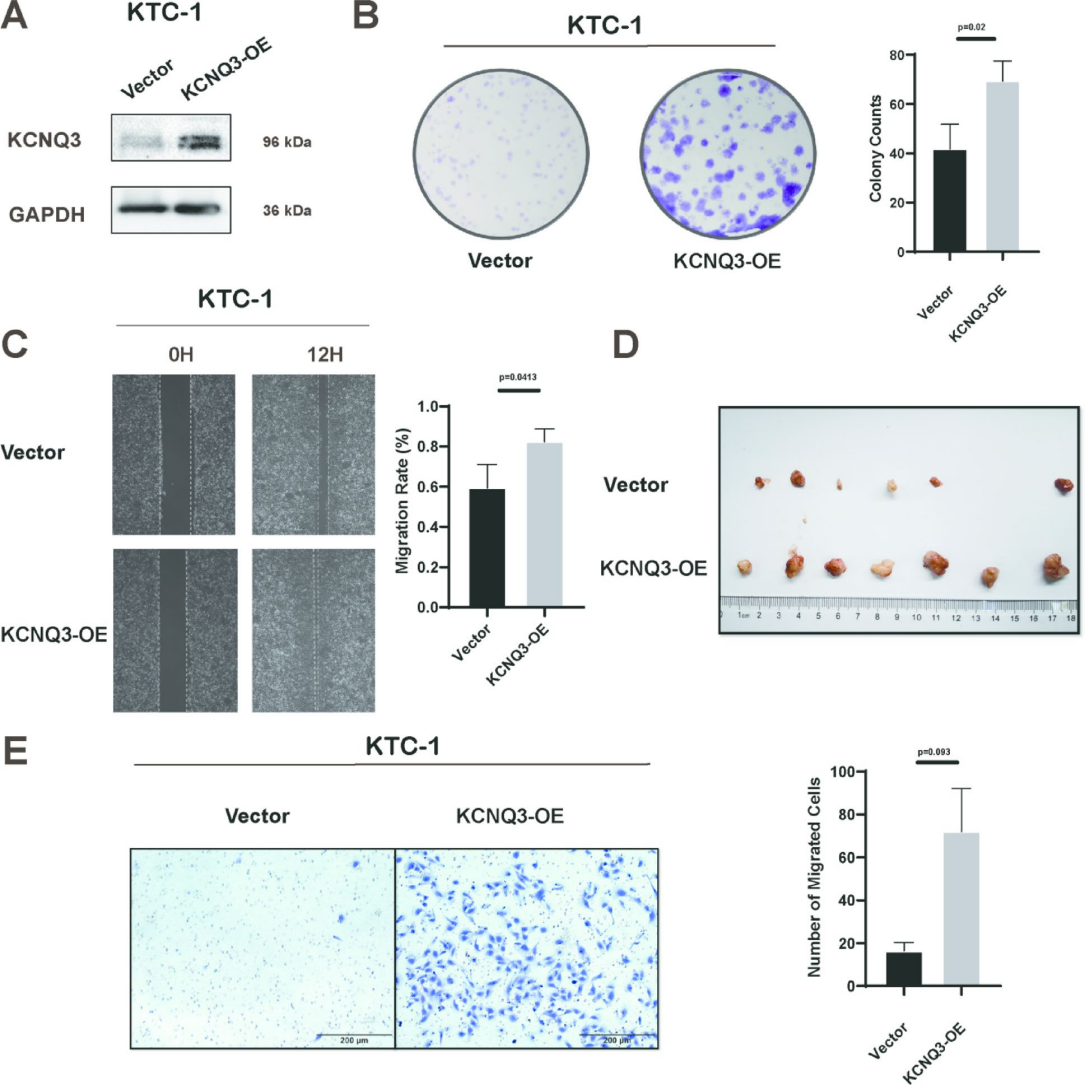
KCNQ3 enhances PTC cell proliferation and migration in vitro and *in vivo.* (**A**) Western blot images showing KCNQ3 overexpression in KTC-1 cells. (**B**) Colony formation assay showing increased cell proliferation of KTC-1 cells after KCNQ3 overexpression. (**C**) Wound-healing assay showing enhanced cell migration of KTC-1 cells after KCNQ3 overexpression. (**D**) Tumors that were removed from nude mice. (**E**) Transwell assay showing increased cell migration in KTC-1 cells after KCNQ3 overexpression. Statistical significance of all the data was determined via two-tailed Student’s t-tests. Data are shown as the means ± S.D., *n* = 3

We both knocked down and overexpressed KCNQ3 to assess its influence on PTC cells. KCNQ3 knockdown in high-expressing cell lines resulted in decreased cell proliferation and migration (Fig. 2A–D). Conversely, KCNQ3 overexpression in KTC-1 cells resulted in increased proliferation and migration (Fig. 3A–E). These findings remained consistent in both in vitro and in vivo experiments, as evidenced by the increase and decrease in tumor size in nude mice bearing KCNQ3-overexpressing and KCNQ3-knockdown tumors, respectively (Figs. 2E and 3D, S1A, B). These results indicate that KCNQ3 enhances PTC proliferation and migration.

### KCNQ3 combines with GAREM1, GRB2, and SOS1 to activate the RAS/RAF/MAPK signaling pathway

**Fig. 4:**
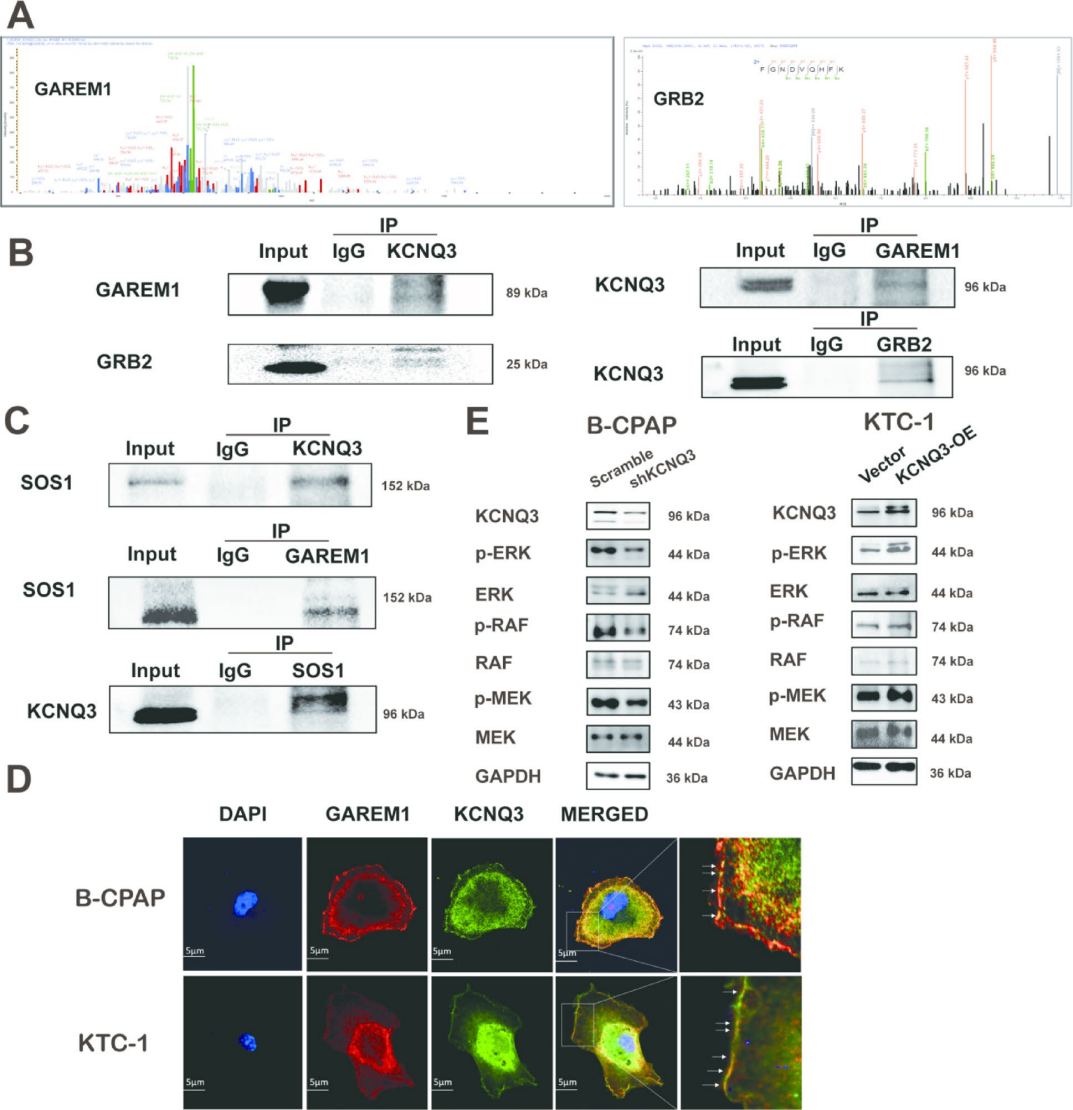
KCNQ3 interacts with GAREM1, GRB2, and SOS1 to activate the RAS/RAF/MAPK signaling pathway. (**A**) Identification of GAREM1 (left) and GRB2 (right) among the KCNQ3-interacting proteins through mass spectrometric analysis. KCNQ3-interacting proteins were immunoprecipitated from B-CPAP cells, followed by MS analysis. (**B**) Co-immunoprecipitation of KCNQ3, GAREM1, and GRB2 in B-CPAP cells. (**C**) Co-immunoprecipitation of KCNQ3, GAREM1, and SOS1 in B-CPAP cells. (**D**) Immunofluorescence of KCNQ3 and GAREM1 in KTC-1 and B-CPAP cells. (**E**) Western blot images showing the protein levels of RAS/RAF/MAPK signaling components after KCNQ3 knockdown or overexpression in PTC cells.

To elucidate the mechanistic role of KCNQ3 in PTC progression, we employed co-immunoprecipitation (co-IP) in B-CPAP cells, followed by mass spectrometry (MS) to identify KCNQ3-interacting partners (Fig. 4A). Our MS results revealed GAREM1 and GRB2 to be key interactors, with subsequent co-IP validating a protein complex comprising KCNQ3, GAREM1, GRB2, and SOS1 (Fig. 4B, C, S2A). Notably, GAREM1 is known to activate the RAS/RAF/MAPK pathway and is implicated in tumor development [23]. Intriguingly, although KCNQ3 normally associates with KCNQ2, overexpressed KCNQ3 in PTC selectively binds GAREM1, promoting its downstream oncogenic functions (Fig. 4B, S2B). Immunofluorescence also corroborated the colocalization of KCNQ3 with GAREM1, reinforcing their functional interplay (Fig. 4D). We hypothesized that this complex drives RAS/RAF/MAPK signaling, a pathway critical for tumorigenesis. Supporting this finding, KCNQ3 knockdown in B-CPAP cells reduced the phosphorylation of RAF, MEK, and ERK, key effectors in the MAPK cascade, whereas KCNQ3 overexpression enhanced their activation (Fig. 4E, S3, S4, S5A, B). These findings, consistently observed across three independent experiments, demonstrate that KCNQ3, through its specific interactions with GAREM1, GRB2, and SOS1, potently activates the RAS/RAF/MAPK pathway, thereby fueling PTC proliferation and progression.

### Estradiol (E_2_) upregulates KCNQ3 expression and ESR1 regulation through KCNQ3 in PTC cell lines

**Fig. 5:**
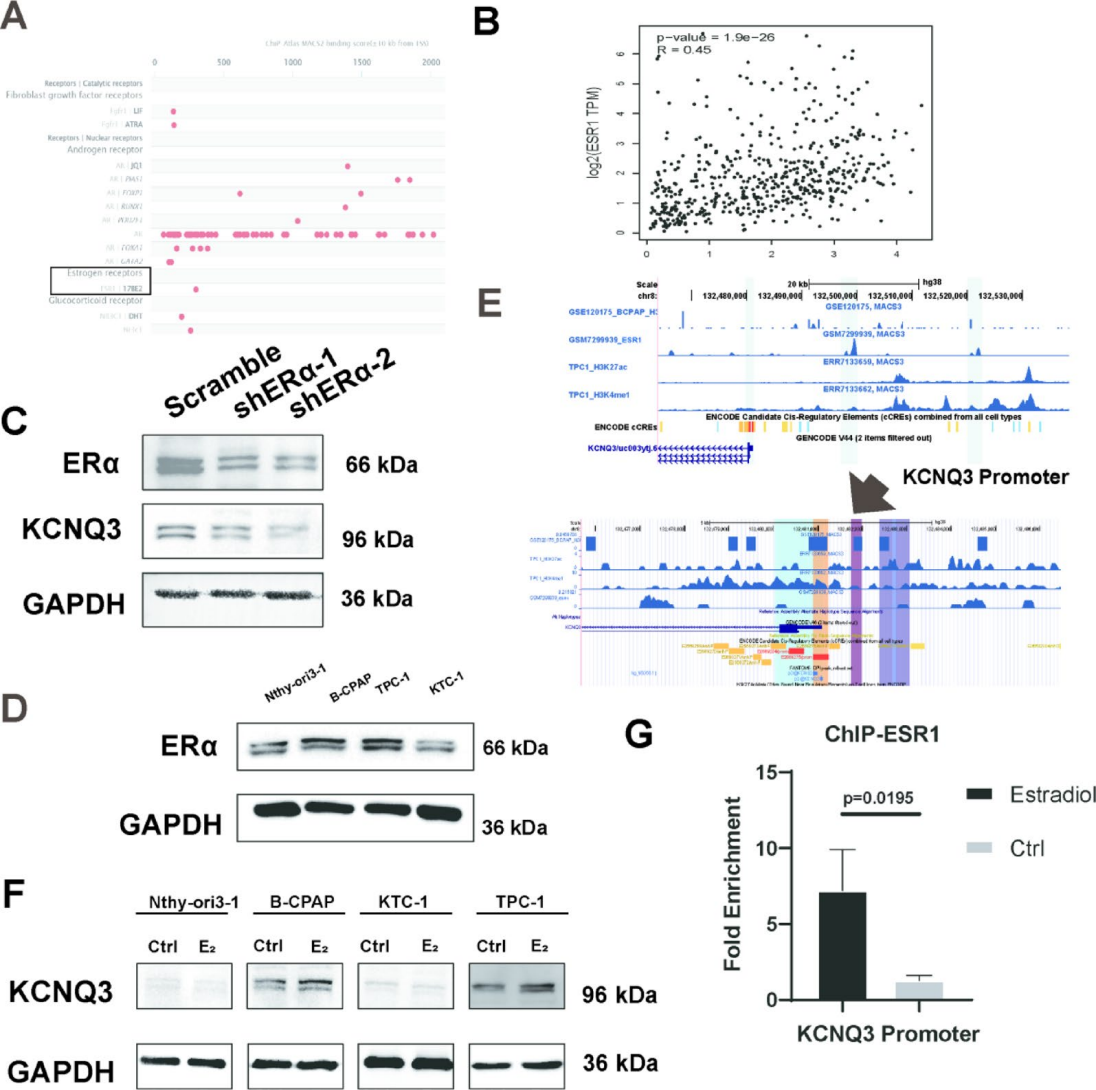
Excessive estradiol increases ESR1 binding to the KCNQ3 promoter region, and increases KCNQ3 transcription. (**A**) Transcription factors regulating KCNQ3 were predicted via the GeneCards database. (**B**) Scatter plot showing the correlation between KCNQ3 and ESR1 expression in the thyroid cancer (THCA) cohort from TCGA, which was analyzed via GEPIA [32]. (**C**) Western blotting showing decreased KCNQ3 expression in ESR1-knockdown B-CPAP cells. (**D**) Western blotting shows the expression level of ESR1 in Nthy-ori3-1 and three PTC cell lines. (**E**) From top to bottom: UCSC gene annotation (GRCh38/hg38) of KCNQ3; enrichment of H3K27ac, ESR1, and H3K4me3 in different cell lines; the “KCNQ3 Promoter” arrow delineates the KCNQ3 promoter. Top: possible binding sites for ESR1 and KCNQ3 enhancers or KCNQ3 promoters. Bottom: KCNQ3 promoter primer loci designed for ChIP‒qPCR to detect ESR1 binding sites in the KCNQ3 promoter region. (**F**) Western blot images showing KCNQ3 expression levels in Nthy-ori3-1 cells and three PTC cell lines with and without E_2_ treatment. (**G**) ESR1 enrichment at the KCNQ3 promoter region in B-CPAP cells was assessed via ChIP‒qPCR. Statistical significance of all data was determined via two-tailed Student’s t-test. Data are shown as mean ± SD, *n* = 3

Given the pronounced female predominance in PTC, with a male-to-female ratio of 1:3 [25], we hypothesized that estrogen signaling drives elevated KCNQ3 expression. Interrogation of the GeneCards database identified ESR1 as a high-confidence transcription factor for KCNQ3 (Fig. 5A), corroborated by a strong correlation between KCNQ3 and ESR1 expression in the GEPIA database (Fig. 5B). To test this, ESR1 was knocked down in B-CPAP cells, resulting in a reduction in KCNQ3 expression, mirroring the predicted ESR1–KCNQ3 relationship (Fig. 5C). To further validate the correlation and explain cell-line variability, we analyzed the basal expression of ESR1 across different thyroid cell lines. Western blot analysis revealed that ESR1 was highly expressed in B-CPAP and TPC-1 cells, but its expression was minimal in KTC-1 and non-malignant Nthy-ori3-1 cells (Fig. 5D). This differential ESR1 expression pattern closely paralleled the inherent KCNQ3 expression levels observed in these lines (Fig. 1D). Our statistical analysis also showed a correlation between ESR1 and KCNQ3 (Table S1). In addition, we analyzed the ChIP-seq data of ESR1 and histone modifications for the PTC lines TPC-1 and B-CPAP, and designed primers to assess the binding of ESR1 to the KCNQ3 promoter region (Fig. 5E). Stimulation with 10⁻⁸ M estradiol (E_2)_ upregulated KCNQ3 expression in B-CPAP cells, derived from a female patient, but not in KTC-1 cells, originating from a male patient [33], nor in TPC-1 cells, which lack E_2_ sensitivity despite a female origin [34, 35](Fig. 5F, S5C). ChIP–qPCR with the primers mentioned above further revealed enhanced ESR1 occupancy at the KCNQ3 promoter in E_2_-stimulated B-CPAP cells (Fig. 5G). To confirm the specificity of ESR1 mediation, we utilized the selective estrogen receptor antagonist, fulvestrant (1 µM). In B-CPAP cells, co-treatment with fulvestrant abrogated the E_2_-induced upregulation of KCNQ3 expression (Fig. S6). To investigate whether E_2_ stimulation can activate the GAREM1–GRB2–SOS1–MAPK signaling pathway under KCNQ3 knockdown, we conducted a KCNQ3 immunoprecipitation (IP) experiment in B-CPAP cells. B-CPAP cells, transfected with either a scramble control or shKCNQ3, were treated with or without E_2_, followed by IP to pull down KCNQ3 and probe for GAREM1.The IP results revealed an obvious GAREM1 band that co-immunoprecipitated with KCNQ3 in the non-knockdown (scramble) group, whereas both the shKCNQ3 group and the E_2_-treated shKCNQ3 group exhibited minimal GAREM1 pulldown (Fig. S7). These results demonstrate that E_2_ specifically modulates KCNQ3 transcription to facilitate KCNQ3-GAREM1 binding, thereby activating the KCNQ3-GAREM1–GRB2–SOS1–MAPK signaling pathway in PTC. Collectively, these data establish that E_2_, via ESR1-mediated transcriptional activation, potently upregulates KCNQ3 expression, elucidating a molecular basis for sex-specific KCNQ3 dysregulation in PTC.

### XE991 inhibits PTC cell proliferation and migration

**Fig. 6:**
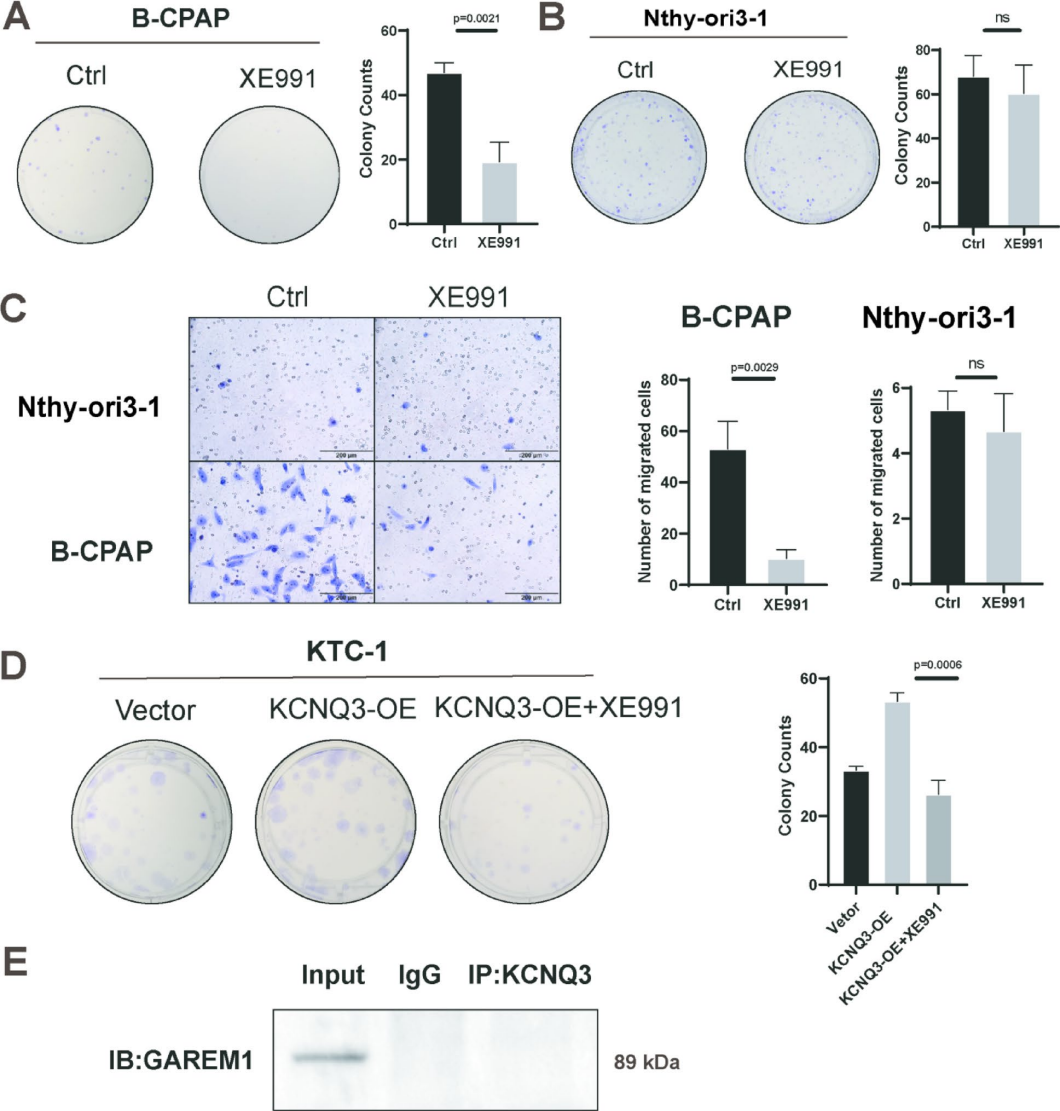
XE991 inhibits PTC cell proliferation and migration. (**A**) Colony formation assay showing B-CPAP cell proliferation inhibition after treatment with XE991 (10 µM). (**B**) Colony formation assay showing no change in Nthy-ori3-1 cell proliferation after treatment with XE991. (**C**) Transwell assay showing inhibition of B-CPAP cell migration and invasion after treatment with XE991, whereas no change was observed in Nthy-ori3-1 cells. (**D**) Colony formation assay showing inhibition of cell proliferation by XE991 in KCNQ3-overexpressing KTC-1 cells. (**E**) Co-immunoprecipitation showing a loss of KCNQ3 and GAREM1 association in B-CPAP cells after treatment with XE991. Statistical significance of all the data was determined via two-tailed Student’s t-tests. Data are shown as mean ± SD, *n* = 3

Given the pivotal role of KCNQ3 in PTC progression, we explored targeted therapeutic strategies to mitigate its oncogenic activity. We identified XE991 (10 µM), a selective KCNQ channel inhibitor, as a candidate drug to suppress KCNQ3 function in PTC [29, 31]. Functional assays revealed that XE991 potently inhibited the proliferation and migration of B-CPAP cells, with minimal effects on non-malignant Nthy-ori3-1 thyroid cells, underscoring its tumor-specific efficacy (Fig. 6A–C). In KTC-1 cells engineered to overexpress KCNQ3, XE991 effectively reversed KCNQ3-driven hyperproliferation, further validating its therapeutic potential (Fig. 6D). To elucidate the underlying mechanism, co-immunoprecipitation analysis of XE991-treated B-CPAP cells displayed a striking disruption of the KCNQ3–GAREM1 interaction, a critical driver of RAS/RAF/MAPK signaling (Fig. 6E). To establish the therapeutic concentration range and confirm its dose-dependent efficacy, we performed a time- and dose-dependent cell viability assay on B-CPAP cells (Fig. S8). The results demonstrate that XE991 inhibits cell viability in a clearly dose-dependent manner across the concentrations tested. Specifically, 10 µM XE991 significantly suppressed cell growth at the 36-hour and 48-hour time points, consistent with our subsequent functional assays. Furthermore, the 30 µM and 50 µM doses exhibited a stronger inhibitory effect, confirming the concentration-dependent nature of its action and a favorable specificity and therapeutic window at the selected therapeutic concentration (10 µM).

## Discussion

**Fig. 7:**
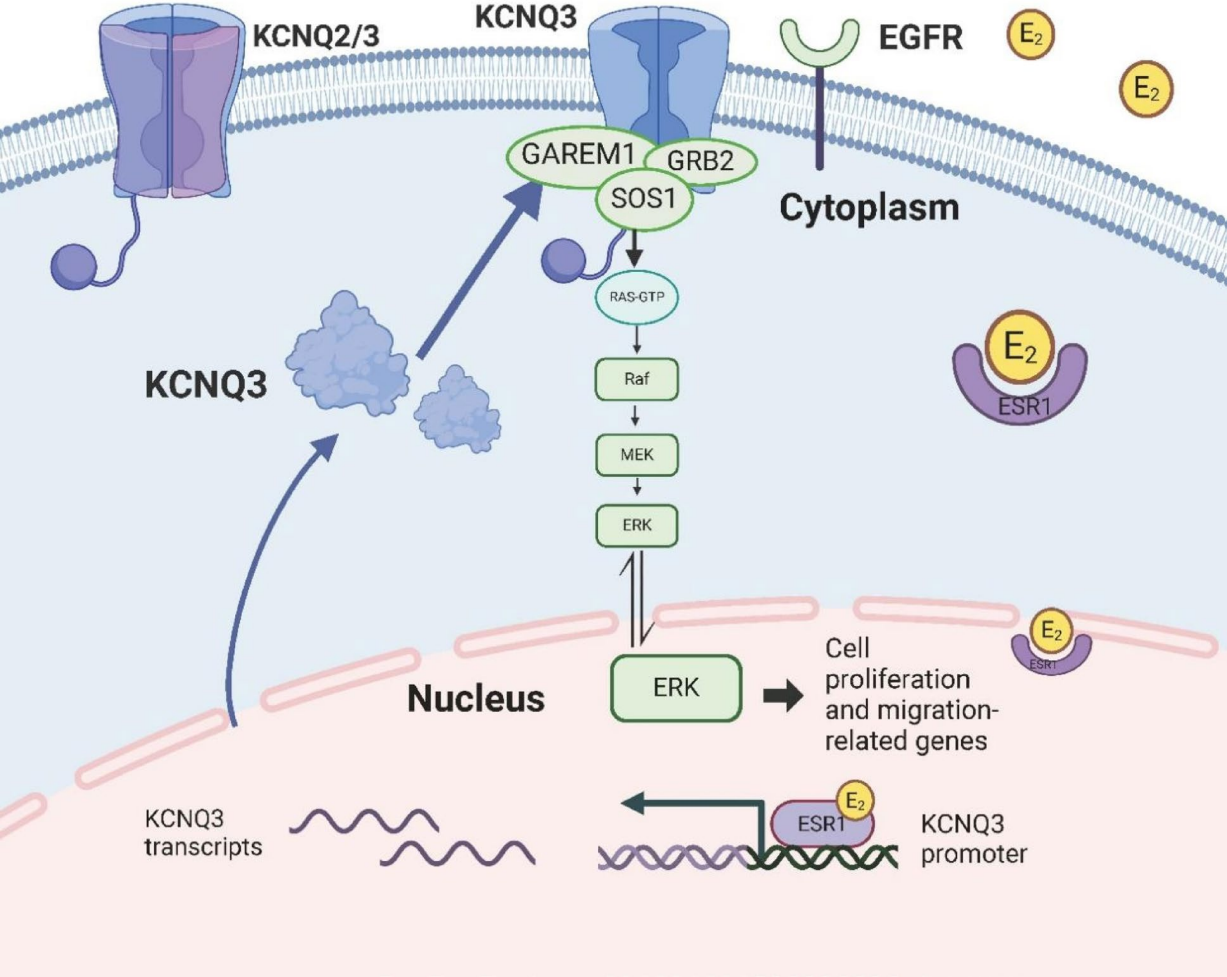
Schematic diagram illustrating the mechanism by which KCNQ3 enhances tumor progression. Estradiol enters the cell from the extracellular environment and binds to the estrogen receptor (ESR1) located in the cytoplasm. The receptor-ligand complex translocates to the nucleus and interacts with the KCNQ3 promoter region, promoting KCNQ3 transcription. Upregulated KCNQ3 expression enables its interaction with GAREM1, GRB2, and SOS1 at the inner membrane, forming a complex that activates the RAS/RAF/MAPK signaling pathway. This activation supports downstream signaling mechanisms, contributing to the proliferation and migration of PTC cells

The KCNQ3 potassium channel, traditionally recognized for its role in forming heteromeric channels with KCNQ2 to regulate neuronal excitability, has emerged as a pivotal regulator in the pathogenesis of PTC [19–21, 36]. Our study provides compelling evidence that KCNQ3 is significantly upregulated in PTC tissues, driving tumor cell proliferation, migration, and progression through mechanisms independent of KCNQ2. This oncogenic potential is mediated by a novel interaction with GAREM1, GRB2, and SOS1, forming a molecular complex that activates the RAS/RAF/MAPK signaling cascade. In contrast to the canonical EGFR-driven pathway, where EGF-induced phosphorylation of GAREM1 at tyrosine residues 105 and 453 recruits GRB2 to activate SOS1 and RAS [22–24, 38], our findings reveal that KCNQ3 directly engages this signaling axis by facilitating GDP-GTP exchange on RAS, subsequently triggering the downstream activation of RAF, MEK, and ERK, thereby promoting oncogenesis. These observations reposition KCNQ3 as a versatile signaling hub in PTC, with potential implications for both disease understanding and therapeutic targeting (Fig. 7).

The pronounced female predominance in PTC, with a male-to-female ratio of approximately 1:3 [25], underscores a critical role for estrogen and estrogen receptor signaling in its pathogenesis [36, 37]. E_2_, as the primary estrogen, exerts pleiotropic effects on thyroid cells by promoting proliferation and differentiation through ESR1-mediated pathways, including activation of PI3K/AKT and ERK1/2 signaling [35], potentially contributing to the higher incidence and aggressiveness of PTC in women.

Previous studies have linked E_2_ to the upregulation of genes involved in cell cycle regulation and survival in thyroid tissues, suggesting that hormonal fluctuations may heighten PTC risk [38]. Our research extends this framework by identifying KCNQ3 as a novel downstream effector of E_2_/ESR1 signaling. The observed E_2_-induced upregulation of KCNQ3, which is specifically inhibited by the ESR1 antagonist fulvestrant, provides mechanistic clarity, positioning KCNQ3 as a key membrane protein linking hormone-driven proliferation to sustained oncogenic activity in PTC. This sex-specific regulation is further evidenced by our findings in B-CPAP cells (derived from a female patient), where E_2_ treatment markedly upregulated KCNQ3 expression, correlating with enhanced thyroid cell activity. In contrast, KTC-1 cells (from a male patient whose tumor lacked E_2_ sensitivity) showed no such response, likely reflecting variations in ESR1 expression, co-regulator availability, or epigenetic modifications [35, 39, 40]. Although the KCNQ3 gene promoter lacks a consensus estrogen response element, large-scale data analysis and our experimental results confirm that ESR1 binds to the KCNQ3 promoter. This binding likely occurs indirectly via a non-ERE-dependent tethering pathway, potentially involving cooperative interactions with other nuclear factors, that may underlie the observed regulation, and warrants further exploration to elucidate the precise molecular interactions [43, 44].

The most significant advance from this study lies in elucidating the mechanistic interplay between KCNQ3, GAREM1, GRB2, SOS1, and estrogen signaling in driving PTC progression. We identify over-expressed KCNQ3 as a triggering protein that, through its association with GAREM1, facilitates the assembly of a KCNQ3-GAREM1-GRB2-SOS1 complex, a critical determinant for RAS activation and subsequent RAS/RAF/MAPK cascade activation. Crucially, the E_2_/ESR1-mediated enhancement of KCNQ3 transcription establishes a regulatory loop whereby E_2_ upregulates KCNQ3 expression, forming a novel KCNQ3-GAREM1-GRB2-SOS1 complex that amplifies RAS/RAF/MAPK signaling, providing a molecular mechanism for the enhanced proliferation observed in female patients with PTC. This integrative model highlights how genetic drivers and hormonal factors converge on KCNQ3, offering a comprehensive framework for understanding PTC progression.

From a translational perspective, our findings hold substantial promise. The association of KCNQ3 with advanced clinical stages positions it as a potential biomarker for identifying patients at high risk of recurrence, a critical step toward personalized medicine in PTC management. Furthermore, the demonstrated dose-dependent efficacy of XE991, a selective KCNQ channel inhibitor, to suppress PTC cell proliferation, by disrupting KCNQ3-GAREM1 interaction that abrogates the KCNQ3-GAREM1-GRB2-SOS1 complex, offers a novel therapeutic avenue distinct from traditional kinase inhibitors. However, several limitations temper these findings: the reliance on in vitro cell line and xenograft models necessitates validation in larger preclinical and clinical cohorts; the precise binding dynamics and structure-function relationships of the KCNQ3-GAREM1 complex require high-resolution structural studies; and the indirect nature of E_2_/ESR1 regulation via non-ERE mechanisms call for additional functional assays.

## Conclusion

In this study, we elucidate a novel oncogenic role for KCNQ3 in PTC, demonstrating its significant upregulation in tumor tissues and its association with poor prognosis. Our findings reveal that KCNQ3 promotes PTC cell proliferation and migration, both in vitro and in vivo, through a newly identified molecular mechanism involving the formation of a protein complex with GAREM1, GRB2, and SOS1. This complex activates the RAS/RAF/MAPK signaling pathway, a critical driver of oncogenesis, highlighting KCNQ3 as a key PTC progression regulator. Additionally, we establish that E_2_ enhances KCNQ3 expression via binding ESR1 to the KCNQ3 promoter, providing a molecular explanation for the higher incidence of PTC in females. Therapeutically, the KCNQ channel inhibitor XE991 suppresses PTC cell proliferation and migration, positioning KCNQ3 as a promising target for intervention. These insights significantly advance our understanding of PTC pathogenesis and suggest novel strategies for targeted therapy.

## Supplementary Information

Supplementary Material 1.

## Data Availability

No datasets were generated or analysed during the current study.

## Abbreviations

ChIP: Chromatin immunoprecipitation
ChIP-seq: Chromatin immunoprecipitation sequencing
Co-IP: Co-immunoprecipitation
ddH2O: Double-distilled water
DAPI: 4’,6-Diamidino-2-phenylindole
E_2_: Estradiol
ESR1: Estrogen receptor 1
FBS: Fetal bovine serum
GEPIA: Gene Expression Profiling Interactive Analysis
GRB2: Growth factor receptor-bound protein 2
GAREM1: GRB2-associated regulator of MAPK1 subtype 1
HEK293T: Human embryonic kidney 293T cells
KCNQ3: Potassium voltage-gated channel subfamily Q member 3
LC-MS: Liquid chromatography–mass spectrometry
MAPK: Mitogen-activated protein kinase
PBS: Phosphate-buffered saline
PBST: PBS with Tween 20
PTC: Papillary thyroid cancer
PVDF: Polyvinylidene difluoride
qPCR: Quantitative polymerase chain reaction
RPMI 1640: Roswell Park Memorial Institute 1640 medium
RT–qPCR: Reverse transcription quantitative polymerase chain reaction
SDS-PAGE: Sodium dodecyl sulfate–polyacrylamide gel electrophoresis
shRNA: Short hairpin RNA
SOS1: SOS Ras/Rac guanine nucleotide exchange factor 1
TCGA: The Cancer Genome Atlas
