# Supplementary material for: Increased KCNQ3 expression in papillary thyroid cancer promotes proliferation and migration
Source: Cancer Cell Int. 2025 Nov 17;25:406. doi: 10.1186/s12935-025-04049-6 (PMC12625505; doi:10.1186/s12935-025-04049-6)
Supplement: Supplementary file 1 — Supplementary Material 1. [file 12935_2025_4049_MOESM1_ESM.pdf]

Supplementary Table S1:

Relationship Between KCNQ3 Expression and Clinical Characteristics in Papillary thyroid cancer Patients

	KCNQ3 expression				
**Characteristics**	All patients	Low or no expression (%)	High expression (%)	Pearson χ^2	**P value**
**Total samples**				13.1630	**0.0003 ***
**- normal tissue**	45	35 (77.78%)	10 (22.22%)		
**- cancer tissue**	45	17 (37.78%)	28 (62.22%)		
**Sex**				3.9707	**0.0463 ***
**- Male**	8	6 (75.0%)	2 (25.0%)		
**- Female**	37	12 (29.7%)	25 (70.3%)		
**Age at diagnosis, y**				2.8571	**0.0910**
**- < 50**	15	8 (53.3%)	7 (46.70%)		
**- > 50**	30	9 (30.0%)	21 (70.0%)		
**Tumor grade**				5.9571	**0.0147 ***
**- T0 - T1**	20	12 (60.0%)	8 (40.0%)		
**- T2 - T4**	25	5 (20.0%)	20 (80.0%)		
**Lymph node metastasis**				4.8000	**0.0285 ***
**- Negative**	15	9 (60.0%)	6 (40.0%)		
**- Positive**	30	8 (26.7%)	22 (73.3%)		
**Molecular type**				8.1278	**0.0044 ***
**- ERα +**	31	7 (22.6%)	24 (77.4%)		
**- ERα -**	14	10 (71.4%)	4 (28.6%)		
**Chemotherapy**				0.2143	**0.6434**
**- Yes**	3	2 (66.7%)	1 (33.3%)		
**- No**	42	15 (35.7%)	27 (64.3%)		

*$P < 0.05$

Supplementary Table S2:

Primary antibodies for western blotting (WB), chromatin immunoprecipitation (ChIP), and co-immunoprecipitation (Co-IP).

Antibody	Dilution	Catalog number	Company
KCNQ3	1:300 (WB)<br>3 μg (IP)	APC-051	Alomone Labs
GAREM1	1:1000 (WB)<br>3 μg (IP)	PA5-20845	Thermo Fisher Scientific
GRB2	1:300 (WB)<br>3 μg (IP)	sc-8034	Santa Cruz
KCNQ2	1:300 (WB)<br>3 μg (IP)	sc-271852	Santa Cruz
RAF	1:300 (WB)	sc-7267	Santa Cruz
p-RAF	1:300 (WB)	sc-271929	Santa Cruz
ESR1	1:300 (WB)<br>3 μg (IP)	sc-8002	Santa Cruz
MEK	1:300 (WB)	sc-6250	Santa Cruz
p-MEK	1:300 (WB)	sc-271914	Santa Cruz
SOS1	1:1000 (WB)<br>3 μg (IP)	55041-1-AP	Proteintech
p44/42 MAPK (Erk1/2)	1:1000 (WB)	9102S	Cell Signaling Technology
p-p44/42 MAPK (Erk1/2)	1:1000 (WB)	4370S	Cell Signaling Technology
c-MYC	1:1000 (WB)	18583S	Cell Signaling Technology
p-c-Myc	1:300 (WB)	sc-377552	Santa Cruz
Anti-VEGF receptor 1	1:1000 (WB)	ab32152	Abcam
Cyclin D1	1:5000 (WB)	82681-1-RR	Proteintech
GAPDH	1:20000 (WB)	60004-1-Ig	Proteintech
β-Actin	1:20000 (WB)	66009-1-Ig	Proteintech
Normal rabbit IgG	1 μg (IP)	98136-1-RR	Proteintech
Normal mouse IgG	1 μg (IP)	B900620	Proteintech
Secondary antibodies			
HRP-conjugated goat anti-rabbit IgG(H+L)	1:2000 (WB)	SA00001-2	Proteintech
HRP-conjugated goat anti-mouse IgG(H+L)	1:2000 (WB)	SA00001-1	Proteintech

Supplementary Table S3:

ShRNA sequence list.

shRNA	Sequence
shKCNQ3-1	5′ -GGTTGCTGTGGGAAACCAAGG-3′
shKCNQ3-2	5′ -GCATGAAAGCCTACGCTTTCT-3′
shKCNQ3-3	5′ -GGTGCAGGTCACGGAGTATTA-3′
shESR1#1	5′ -CTACAGGCCAAATTCAGATAA-3′
shESR1#3	5′ -GCTAGAGATCCTGATGATTGG-3′
shNC (scrambled shRNA)	5′ -CCTAAGGTTAAGTCGCCCTCG-3′

Supplementary Figure S1: KCNQ3 knockdown inhibits PTC cell proliferation *in vivo*

A

Scramble

shKCNQ3

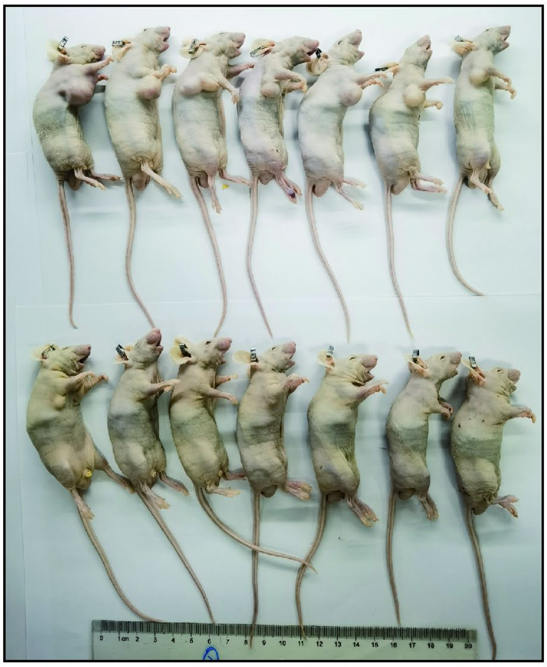

B

Scramble

shKCNQ3

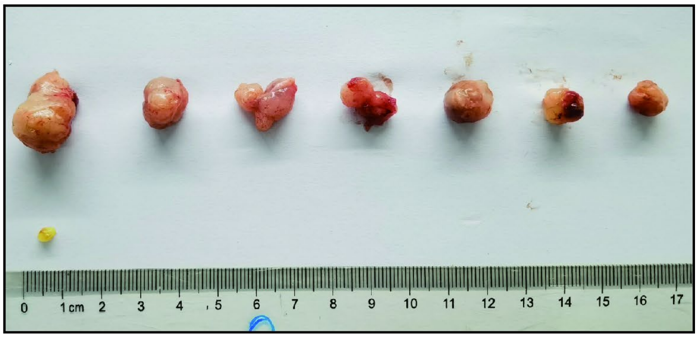

Figure S1. (A). Each nude mouse was subcutaneously injected on their back with 5×10^6 B-CPAP cells in 100 μl Matrigel. After 34 d, all mice were euthanized, and their tumors were excised. (B). Tumors were removed from mice.

Supplementary Figure S2: Co-immunoprecipitation of KCNQ3, GAREM1, and KCNQ2 in B-CPAP cells.

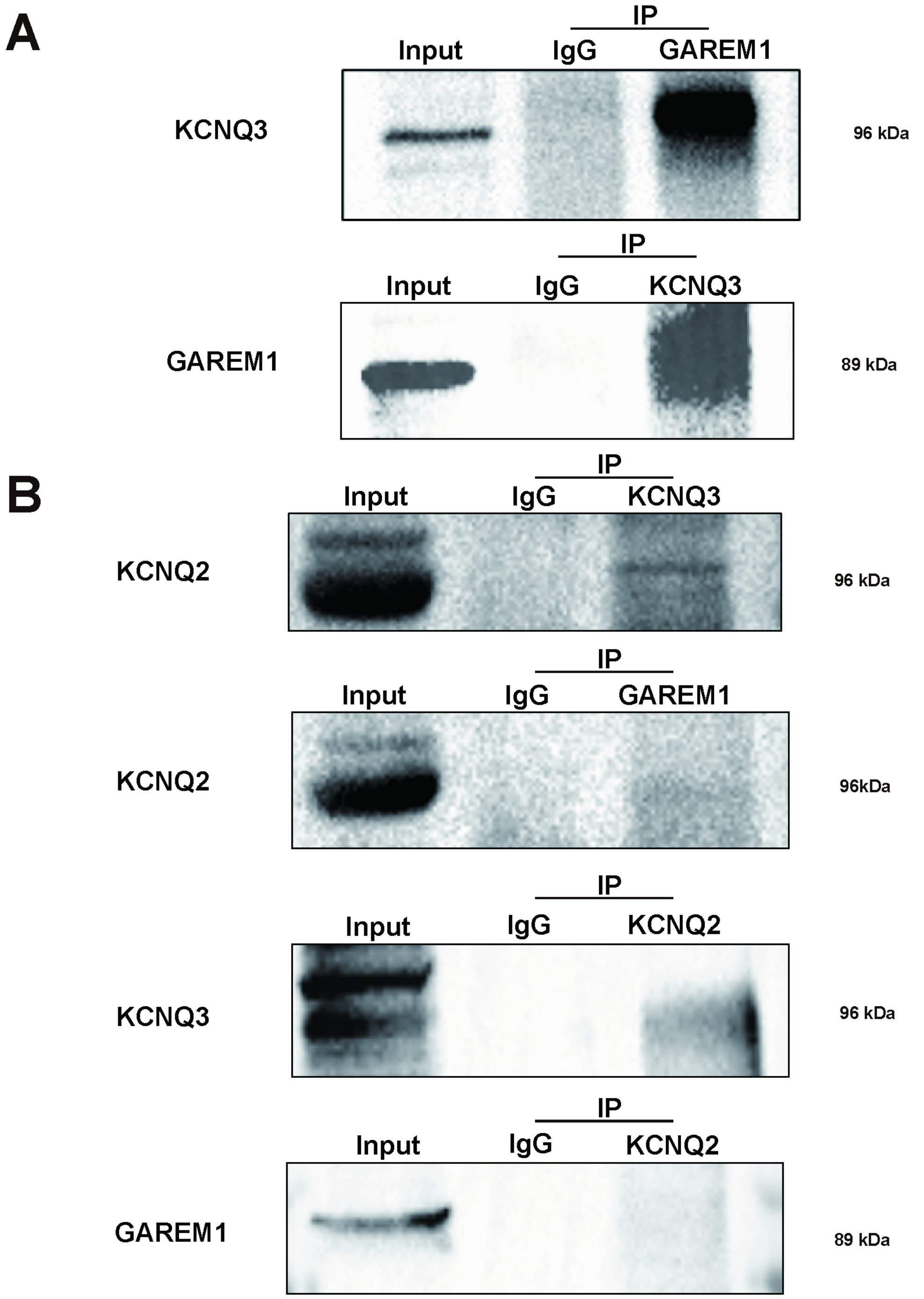

Figure S2. Co-immunoprecipitation shows that KCNQ3 instead of the KCNQ3/KCNQ2 complex activates the RAS/RAF/MAPK signaling pathway through its interaction with GAREM1, GRB2, and SOS1in PTC cells.

Supplementary Figure S3: Western blots showing protein levels of RAS/RAF/MAPK signaling components after KCNQ3 knockdown in B-CPAP cells or overexpression in KTC-1 cells

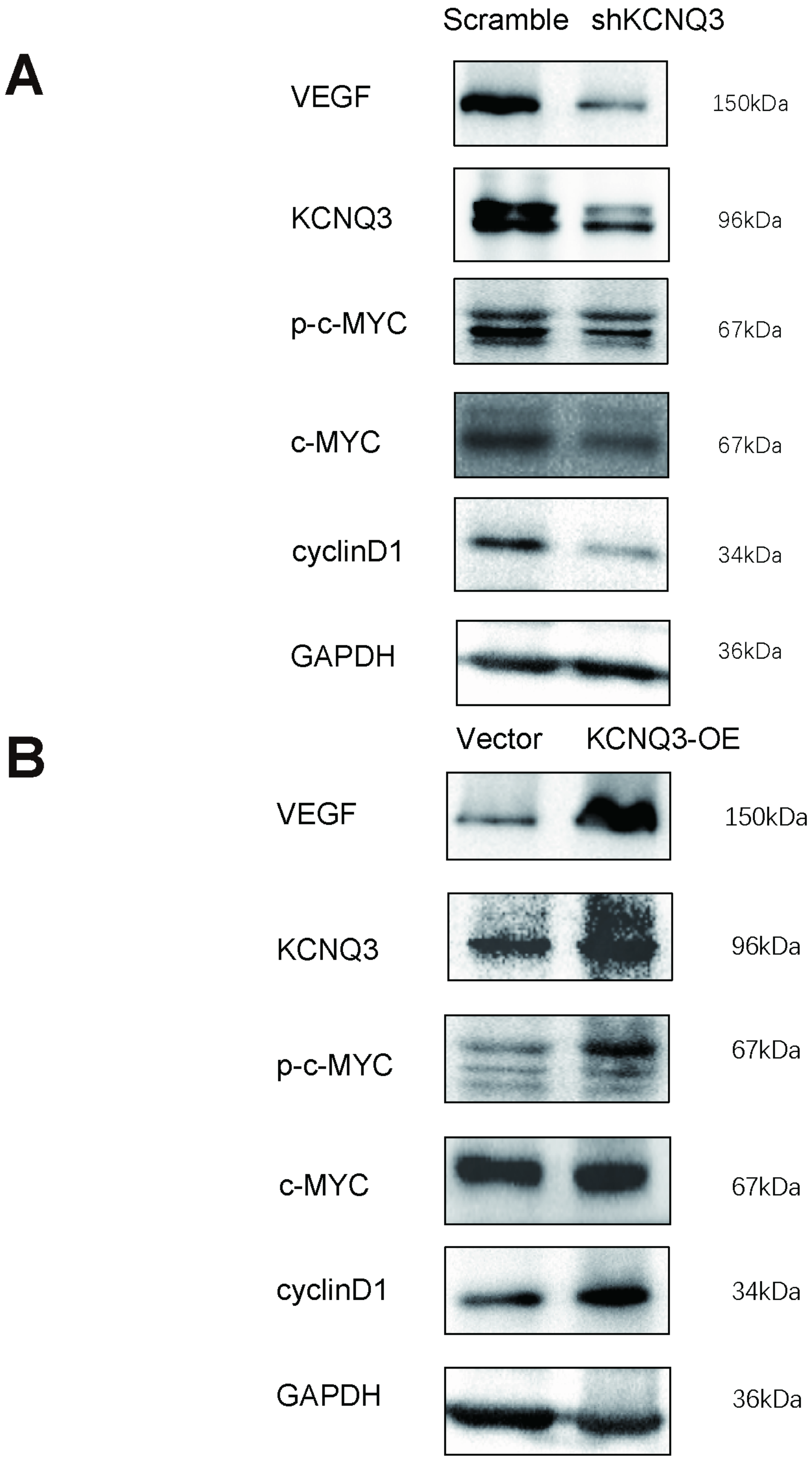

Figure S3. Western blots showing protein levels of RAS/RAF/MAPK signaling components after KCNQ3 knockdown in B-CPAP cells or overexpression in KTC-1 cells.

Supplementary Figure S4:

Kyoto Encyclopedia of Genes and Genomes (KEGG) signaling pathway analysis of KCNQ3 and pathway enrichment of KCNQ3

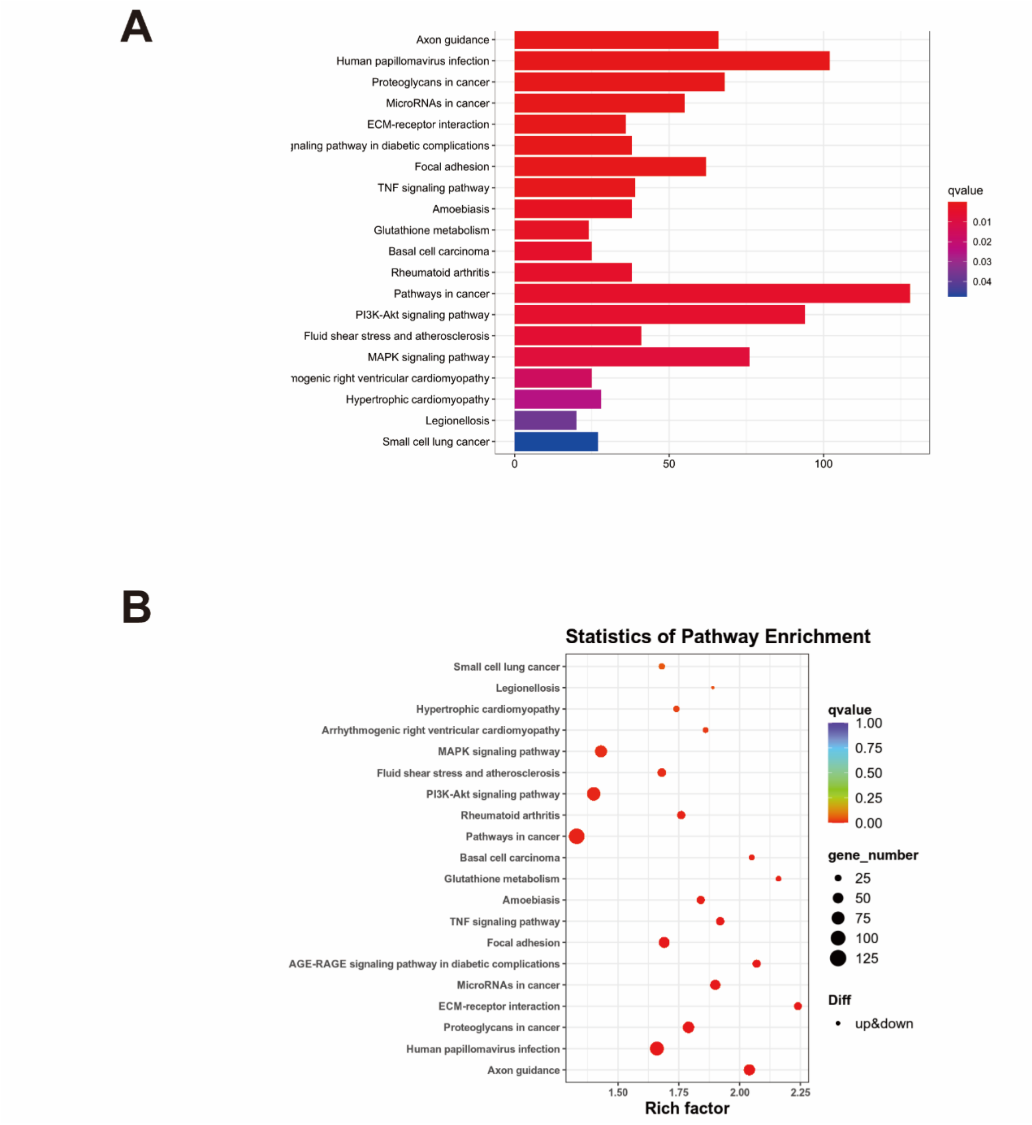

Figure S4. (A)-(B) Kyoto Encyclopedia of Genes and Genomes (KEGG) signaling pathway analysis of KCNQ3 and pathway enrichment of KCNQ3.

Supplementary Figure S5:

Quantification of western blots showing the protein levels of RAS/RAF/MAPK signaling components after KCNQ3 knockdown or overexpression, and the impact of E_2 treatment on KCNQ3 protein expression in thyroid cell lines

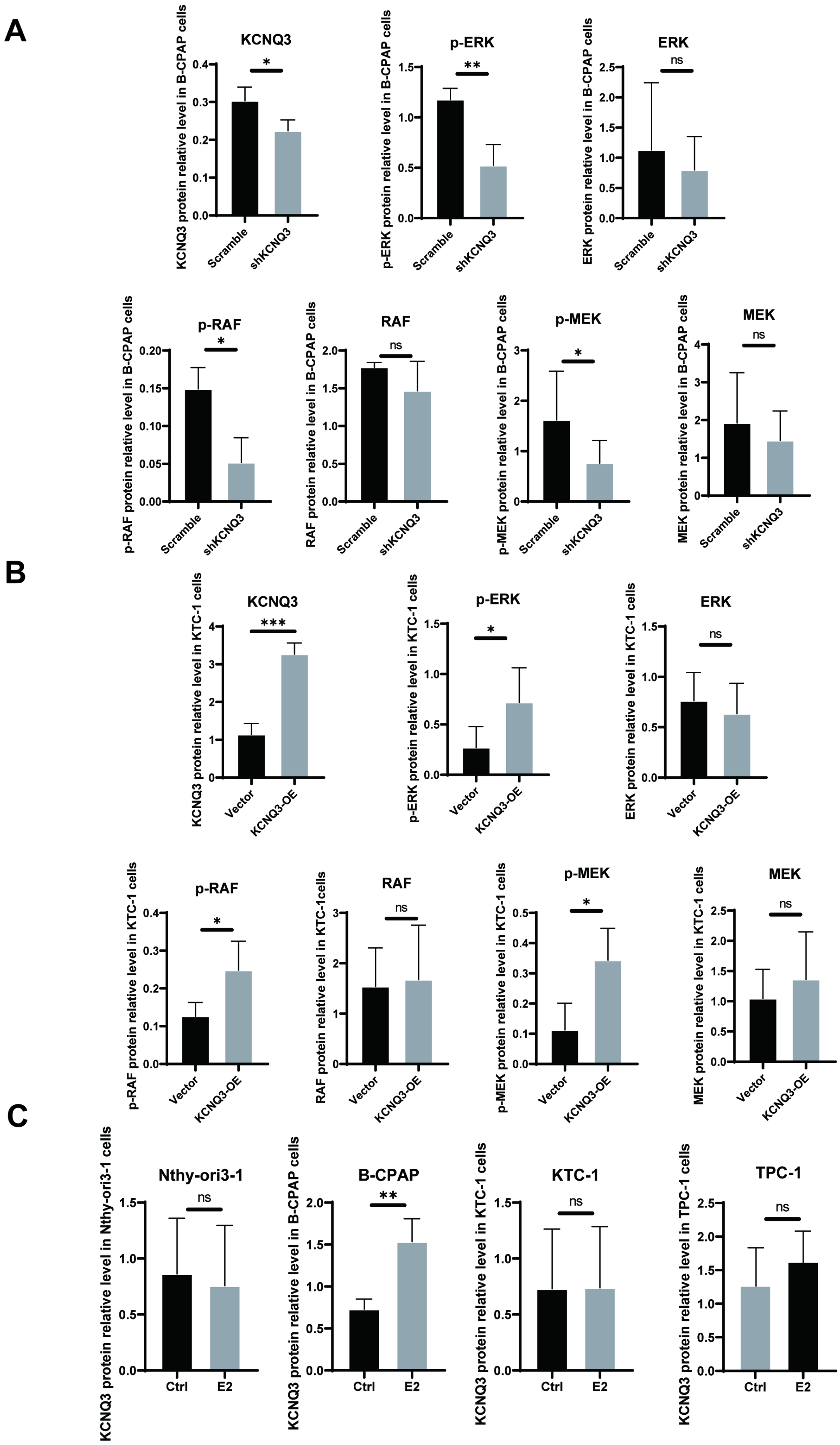
A
KCNQ3
*
KCNQ3 protein relative level in B-CPAP cells
0.0
0.1
0.2
0.3
0.4
Scramble
shKCNQ3
p-ERK
**
p-ERK protein relative level in B-CPAP cells
0.0
0.5
1.0
1.5
Scramble
shKCNQ3
ERK
ns
ERK protein relative level in B-CPAP cells
0.0
0.5
1.0
1.5
2.0
2.5
Scramble
shKCNQ3
p-RAF
*
p-RAF protein relative level in B-CPAP cells
0.00
0.05
0.10
0.15
0.20
Scramble
shKCNQ3
RAF
ns
RAF protein relative level in B-CPAP cells
0.0
0.5
1.0
1.5
2.0
Scramble
shKCNQ3
p-MEK
*
p-MEK protein relative level in B-CPAP cells
0
1
2
3
Scramble
shKCNQ3
MEK
ns
MEK protein relative level in B-CPAP cells
0
1
2
3
4
Scramble
shKCNQ3
B
KCNQ3
***
KCNQ3 protein relative level in KTC-1 cells
0
1
2
3
4
Vector
KCNQ3-OE
p-ERK
*
p-ERK protein relative level in KTC-1 cells
0.0
0.5
1.0
1.5
Vector
KCNQ3-OE
ERK
ns
ERK protein relative level in KTC-1 cells
0.0
0.5
1.0
1.5
Vector
KCNQ3-OE
p-RAF
*
p-RAF protein relative level in KTC-1 cells
0.0
0.1
0.2
0.3
0.4
Vector
KCNQ3-OE
RAF
ns
RAF protein relative level in KTC-1cells
0
1
2
3
Vector
KCNQ3-OE
p-MEK
*
p-MEK protein relative level in KTC-1 cells
0.0
0.1
0.2
0.3
0.4
0.5
Vector
KCNQ3-OE
MEK
ns
MEK protein relative level in KTC-1 cells
0.0
0.5
1.0
1.5
2.0
2.5
Vector
KCNQ3-OE
C
Nthy-ori3-1
ns
KCNQ3 protein relative level in Nthy-ori3-1 cells
0.0
0.5
1.0
1.5
Ctrl
E2
B-CPAP
**
KCNQ3 protein relative level in B-CPAP cells
0.0
0.5
1.0
1.5
2.0
Ctrl
E2
KTC-1
ns
KCNQ3 protein relative level in KTC-1 cells
0.0
0.5
1.0
1.5
Ctrl
E2
TPC-1
ns
KCNQ3 protein relative level in TPC-1 cells
0.0
0.5
1.0
1.5
2.0
2.5
Ctrl
E2

Figure S5. Quantification of western blots showing the protein levels of RAS/RAF/MAPK signaling components after KCNQ3 knockdown or overexpression, and the impact of E_2 treatment on KCNQ3 protein expression in thyroid cell lines

A. Relative protein levels of KCNQ3, p-ERK, ERK, p-RAF, RAF, p-MEK, and MEK in B-CPAP cells transfected with scramble control (Scramble) or KCNQ3 knockdown plasmid (shKCNQ3). Data represent mean ± SEM from three independent experiments, normalized to GAPDH. Statistical significance was determined by Student's t-test: *p < 0.05, **p < 0.01, ***p < 0.001; ns, not significant.

B. Relative protein levels of KCNQ3, p-ERK, ERK, p-RAF, RAF, p-MEK, and MEK in KTC-1 cells transfected with vector control (Vector) or KCNQ3 overexpression plasmid (KCNQ3-OE). Data represent mean ± SEM from three independent experiments, normalized to GAPDH. Statistical significance: *p < 0.05, ***p < 0.001; ns, not significant.

C. Relative KCNQ3 protein levels in Nthy-ori-3-1, B-CPAP, KTC-1, and TPC-1 cells under control (Ctrl) or E_2 treatment conditions. Data represent mean ± SEM from three independent experiments, normalized to GAPDH. Statistical significance was determined by Student's t-test: *p< 0.05, **p < 0.01, ***p < 0.001; ns, not significant.

Supplementary Figure S6：Fulvestrant inhibits E2-induced KCNQ3 upregulation.

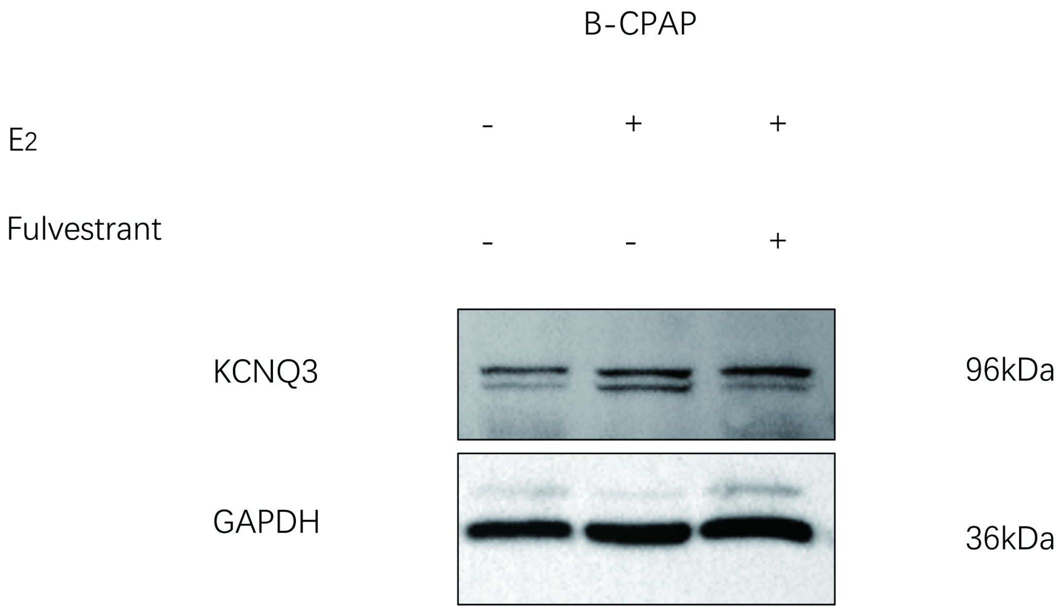

Figure S6.B-CPAP cells were treated with E_2 (10 nM) ± fulvestrant (1 μM). Fulvestrant significantly blocked E_2-induced KCNQ3 expression, confirming ESR1-mediated regulation.

Supplementary Figure S7:

E_2 fails to activate GAREM1–GRB2–SOS1–MAPK signaling under KCNQ3 knockdown.

Figure S7.Co-IP blot showing GAREM1 co-immunoprecipitation with KCNQ3 in scramble control B-CPAP cells, but minimal GAREM1 pulldown in shKCNQ3 and E_2-treated shKCNQ3 groups, indicating E_2's dependence on KCNQ3 for pathway activation in PTC.

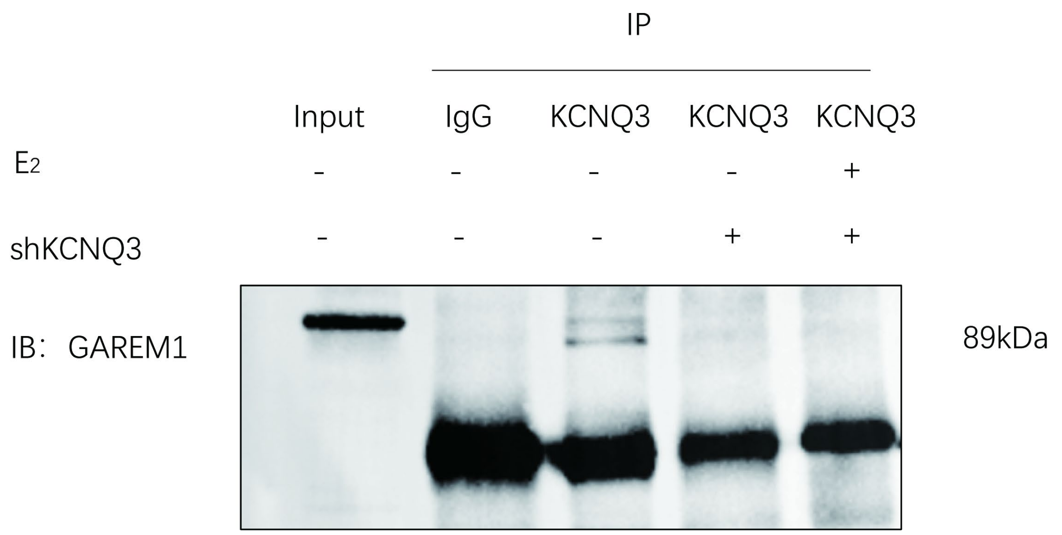

Supplementary Figure S8:

Dose-dependent inhibition of B-CPAP cell viability by XE991.

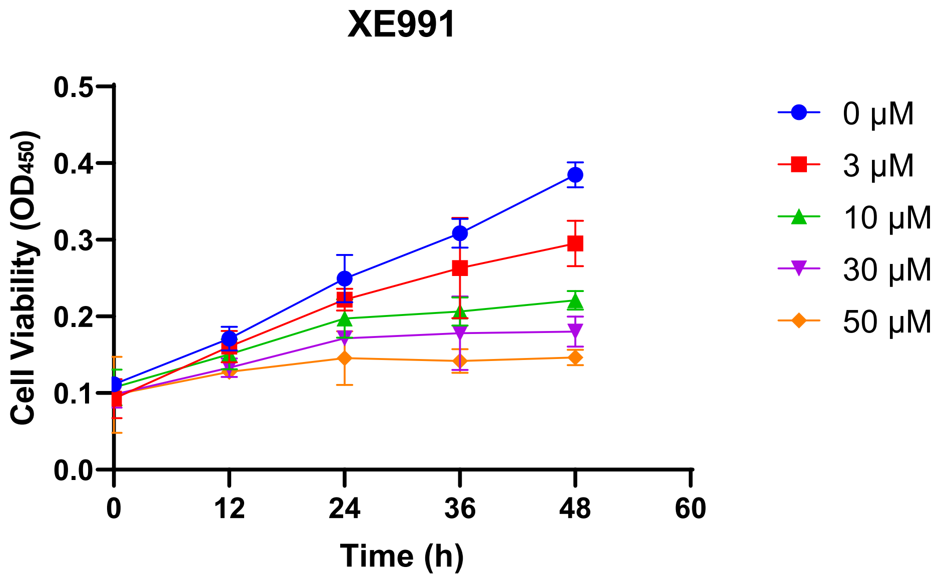

Figure S8. Dose-dependent inhibition of B-CPAP cell viability by XE991. Time- and dose-dependent cell viability assay showing that XE991 (10–50 μM) significantly suppresses B-CPAP cell growth, with 10 μM XE991 reducing cell viability compared to 0 μM (P=0.042017704 at 36 hours; P=0.009861275 at 48 hours) and stronger inhibition at higher concentrations (30 μM and 50 μM) at 36 and 48 hours, confirming its concentration-dependent efficacy and therapeutic window at 10 μM.
